# The cytokine-cosmc signaling axis upregulates the tumor-associated carbohydrate antigen Tn

**DOI:** 10.18632/oncotarget.11324

**Published:** 2016-08-17

**Authors:** Chia-Wen Ho, Chi-Yu Lin, Yi-Wei Liaw, Hsiao-Ling Chiang, Yu-Tang Chin, Rui-Lan Huang, Hung-Cheng Lai, Yaw-Wen Hsu, Po-Jan Kuo, Chiao-En Chen, Hung-Yun Lin, Jacqueline Whang-Peng, Shin Nieh, Earl Fu, Leroy F. Liu, Jaulang Hwang

**Affiliations:** ^1^ Center for Cancer Research, Taipei Medical University, Taipei, Taiwan; ^2^ Department of Biochemistry, Medical College, Taipei Medical University, Taipei, Taiwan; ^3^ Institute of Biochemistry and Molecular Biology, National Yang-Ming University, Taipei, Taiwan; ^4^ Graduate Institute of Cancer Biology and Drug Discovery, College of Medical Science and Technology, Taipei Medical University, Taipei, Taiwan; ^5^ Department of Obstetrics and Gynecology, Shuang-Ho Hospital, Taipei Medical University, New Taipei City, Taiwan; ^6^ Graduate Institute of Life Sciences, National Defense Medical Center, Taipei, Taiwan; ^7^ Department of Obstetrics and Gynecology, School of Medicine, College of Medicine, Taipei Medical University, Taipei, Taiwan; ^8^ Department of Periodontology, School of Dentistry, National Defense Medical Center and Tri-Service General Hospital, Taipei, Taiwan; ^9^ Department of Pathology, National Defense Medical Center and Tri-Service General Hospital, Taipei, Taiwan

**Keywords:** Tn antigen, tumor-associated carbohydrate, cytokines, cosmc, hypermethylation

## Abstract

Tn antigen (GalNAc-α-*O*-Ser/Thr), a mucin-type *O*-linked glycan, is a well-established cell surface marker for tumors and its elevated levels have been correlated with cancer progression and prognosis. There are also reports that Tn is elevated in inflammatory tissues. However, the molecular mechanism for its elevated levels in cancer and inflammation is unclear. In the current studies, we have explored the possibility that cytokines may be one of the common regulatory molecules for elevated Tn levels in both cancer and inflammation. We showed that the Tn level is elevated by the conditioned media of *Hras^G12V^*-transformed-BEAS-2B cells. Similarly, the conditioned media obtained from LPS-stimulated monocytes also elevated Tn levels in primary human gingival fibroblasts, suggesting the involvement of cytokines and/or other soluble factors. Indeed, purified inflammatory cytokines such as TNF-α and IL-6 up-regulated Tn levels in gingival fibroblasts. Furthermore, TNF-α was shown to down-regulate the *COSMC* gene as evidenced by reduced levels of the *COSMC* mRNA and protein, as well as hypermethylation of the CpG islands of the *COSMC* gene promoter. Since Cosmc, a chaperone for T-synthase, is known to negatively regulate Tn levels, our results suggest elevated Tn levels in cancer and inflammation may be commonly regulated by the cytokine-Cosmc signaling axis.

## INTRODUCTION

Tn (GalNAc-α-*O*-Ser/Thr; N-acetyl-galactosamine alpha-*O*-linked to a serine or a threonine residue), a well-known mucin-type *O*-linked glycan, is abnormally overexpressed in a broad spectrum of cancers [1–3]. The expression of Tn antigen often correlates with metastasis and poor prognosis [4]. Previous studies have shown that Tn antigen is an intermediate product in *O*-glycosylation of mucin and can be extended by the key enzyme T-synthase (core 1 β-1, 3-galactosyltransferase or C1β3Gal-T) through transfer galactose from UDP-galactose (uridine diphosphate galactose) to generate T antigen [core 1; Gal-β-(1→3)-GalNAc-α-*O*-Ser/Thr], found in several types of cancer [5]. The substrate specificity and specific molecular chaperon of T-synthase core 1 β3-Gal-T-specific molecular chaperone (Cosmc) have also been demonstrated by previous studies [6–8]. Thus, either a defective T-synthase or a decreased expression of Cosmc could prevent the extension of *O*-linked glycosylation of mucin, resulting in an apparently increased expression of Tn antigen. Moreover, Tn antigen also can be modified with sialic acid by α-2, 6-sialyltransferase (ST6GalNAc I) to produce sTn (sialyl Tn; Neu5Ac-α(2→6)-GalNAc-α-*O*-Ser/Thr), which is also increased in cancer [9–12].

Previous studies have demonstrated that in addition to cancers, Tn is also associated with immune disorders. For example, Tn syndrome is characterized by the detection of Tn antigen on blood cells of all lineages [13]. Tn antigen can be detected on the IgA1 hinge region in some IgA nephropathy patients [14]. Additionally, Tn is known to express in chronic inflammatory tissues such as those from patients with rheumatoid arthritis and osteoarthritis [15]. Moreover, elevated Tn expression has been observed in inflammation-inflicted tissue damage [15–18] and found to be associated with modulation of the host immune response [19, 20]. Although several reports have pointed out that inflammatory cytokines can promote glycan epitope (e.g. sialyl-Lewis(x) antigen) by regulating specific glycosyltransferases [21, 22], the molecular mechanism for elevated expression of Tn antigen in cancer and chronic inflammation remains unclear.

The aim of the current studies is to investigate the molecular mechanism of elevated Tn expression in cancer and inflammation. Our results suggest a possible converging mechanism for elevated Tn expression in cancer and inflammation through cytokine-mediated down-regulation of the *COSMC* gene.

## RESULTS

### Elevated Tn levels in cancer tissues

**Figure 1:**
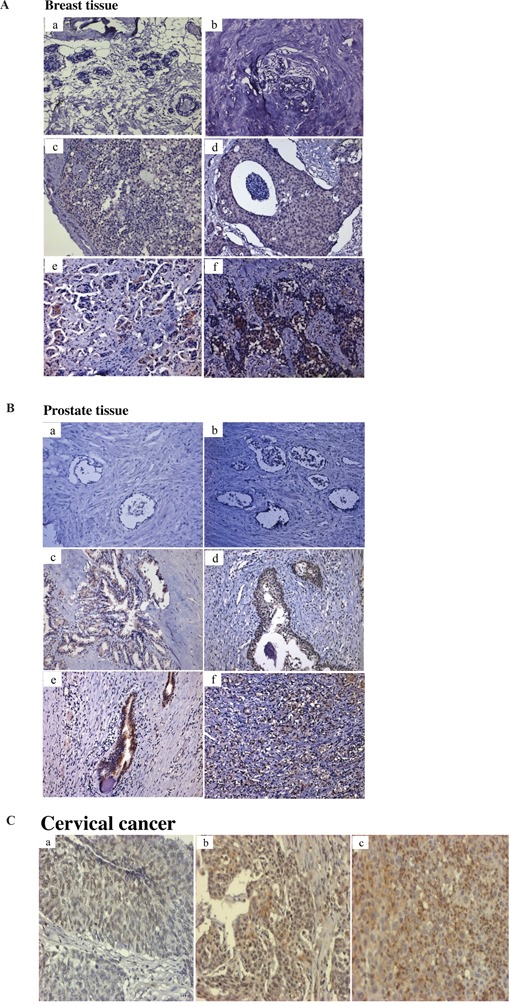
Immunohistochemical analysis of Tn antigen in breast, prostate and cervical tissues **A.** Tn staining in normal breast tiusses specimens (a, b); Tn staining in a grade I (c) and grade III (d) ductal carcinoma in situ (DCIS); Tn staining in a grade I (e) and grade III (f) invasive ductal carcinoma (IDC) **B.** Tn staining in normal prostate tissues specimens (a, b); Tn staining in a Gleason score 6, 7, 8, 9 prostatic adenocarcinomas (c, d, e, f). **C.** Tn staining in a well, moderate and poor differentiation of cervical carcinoma (a, b, c). Brown color represents Tn antigen expression. Original magnifications (100x).

The levels of Tn antigen in breast, prostate and cervical cancers were measured by immunohistochemistry (IHC) as described in Materials and methods. A significant increase in Tn levels were detected in both ductal carcinoma in *situ* (DCIS) and invasive ductal carcinoma (IDC) of breast cancer tissues but not in normal breast tissues (Figure 1A). A significant increase in Tn levels were also observed in prostatic adenocarcinoma but not in normal prostate tissues (Figure 1B). Examination of cervical cancer tissues demonstrated high levels of Tn in moderately to poorly differentiated tumors and lower levels in well differentiated cervical cancer tissues (Figure 1C). Consistent with previous studies, our results indicate that Tn antigen levels are elevated in many cancer tissues.

### Elevated Tn levels in *Hras^G12V^*-transformed cells

**Figure 2:**
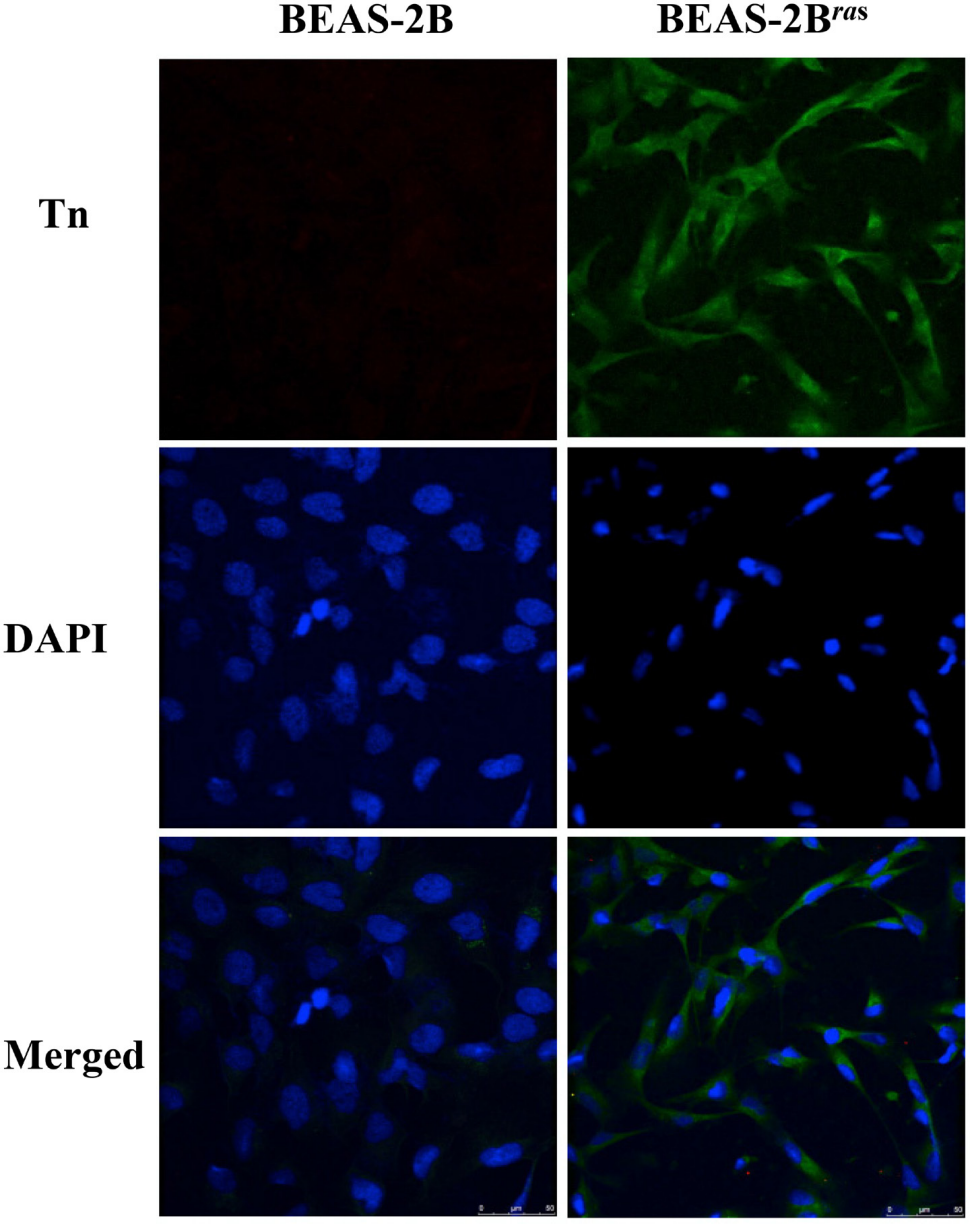
Tn levels is up-regulated in oncogenic Ras-transformed cells Two-days cultured BEAS-2B cells and BEAS-2B*^ras^* cells were immunostained with mouse monoclonal anti-Tn antibody from ascites (in green) and DAPI (in blue).

The possibility that oncogenes may be responsible for elevated Tn levels in cancer was investigated using a pair of *Hras^G12V^*-transformed cell lines, BEAS-2B and *Hras^G12V^*-transformed BEAS-2B (BEAS-2B*^ras^*) cells. As shown in Figure 2, the level of Tn was significantly elevated in BEAS-2B*^ras^* cells, as compared to BEAS-2B cells, when cells were cultured under the same conditions. Thus, induction of a tumorigenic state by oncogenic Ras is associated with an increase in Tn levels.

### Conditioned media from oncogenic Ras-transformed cells and MCF7 breast cancer cells elevate Tn levels

**Figure 3:**
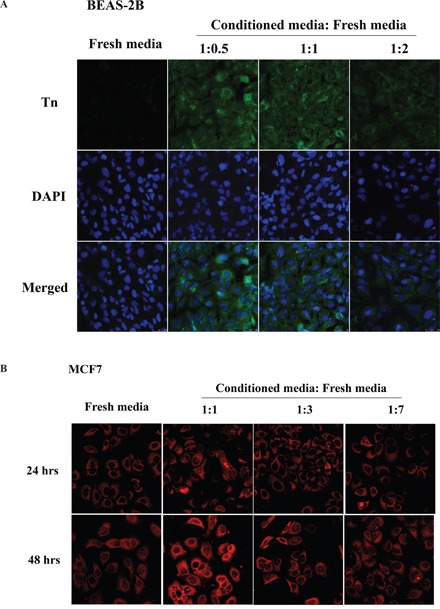
Conditioned media from oncogenic Ras-transformed cells and MCF7 breast cancer cells up-regulate Tn levels **A.** The effect of conditioned media on Tn expression in BEAS-2B cells. The freshly cultured BEAS-2B cells were treated with different dilution ratio of two-days conditioned media obtained from BEAS-2B*^ras^* cells for 48 hours and then stained with mouse monoclonal anti-Tn antibody from ascites (in green) and DAPI (in blue). **B.** The effect of conditioned media on Tn expression in MCF7 cells. The freshly cultured MCF7 cells were treated with different dilution ratio of two-days conditioned media obtained from MCF7 cells for 24 (top) and 48 (bottom) hours and then stained with purified rabbit anti-Tn antibody (in red).

Previous studies have demonstrated that the conditioned media from oncogene-transformed cells can increase the transformation phenotypes and inflammatory cytokines [23]. To test whether the conditioned media can elevate Tn levels, 2-day conditioned media from BEAS-2B*^ras^* cells were collected. The effect of various dilutions of conditioned media in different ratios (1:0.5, 1:1 or 1:2) with fresh culture media on Tn levels in freshly cultured BEAS-2B cells was measured. Significant elevation of Tn levels was observed when BEAS-2B cells were replenished with diluted (1:0.5) conditioned media for 48 hrs, while Tn levels were modestly elevated with more diluted conditioned media (Figure 3A). Similar results were obtained on the effect of conditioned media on Tn levels in MCF7 breast cancer cells (Figure 3B). These results could be explained by the secretion of a Tn-regulatory factor(s) from oncogene-transformed and cancer cells, and the factor(s) could possibly be an inflammatory cytokine(s).

### Elevated Tn levels in inflammatory tissues and cells

**Figure 4:**
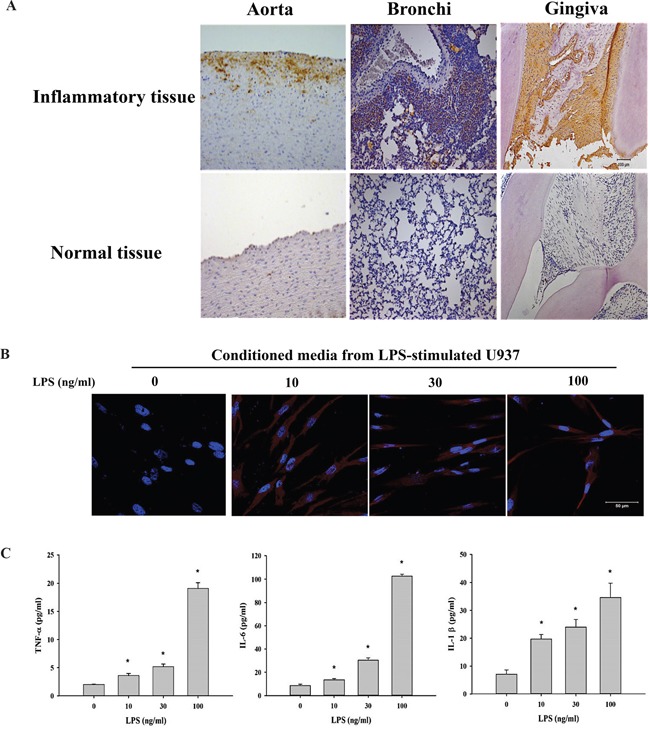
Tn levels is up-regulated in inflammatory tissues and cells **A.** Immunohistochemical analysis of Tn antigen in inflammatory tissues (top) and normal tissues (bottom). Tn staining in atherosclerotic aorta (left; labeled Aorta), bronchitis tissue (middle; labeled Bronchi), and periodontitis tissue (right; labeled Gingiva). Brown color represents Tn antigen expression. **B.** HGFs were treated with conditioned media from U937 cells stimulated with LPS (0, 10, 30 and 100 ng/ml; 24 hours) for 24 hours and then stained with purified rabbit anti-Tn antibody (in red) and DAPI (in blue). **C.** U937 cells were treated with LPS (0, 10, 30 and 100 ng/ml) for 24 hours, the secretions of TNF-α, IL-6 and IL-1β were analyzed by ELISA. Original magnifications (100x). Scale bar, 50 μm.

To examine whether elevated Tn levels are associated with inflammation, Tn levels were measured in inflammatory tissues using immunohistochemistry (IHC). A significant increase in Tn levels was observed in tissues of atherosclerosis, bronchitis and periodontitis but not in their corresponding normal tissues (Figure 4A). To investigate the possible regulation of Tn levels by inflammatory cytokines, conditioned media from monocyte U937 cells stimulated with LPS were used. Tn levels in human gingival fibroblasts (HGFs) replenished with one-day conditioned media from LPS-stimulated U937 cells were observed to increase in an LPS-dose-dependent manner (Figure 4B). The secretion of inflammatory cytokines (ex: TNF-α, IL-6, and IL-1β) was significantly higher in conditioned media from U937 cells treated with LPS (10, 30, or 100 ng/ml) for 24 hours compared with media from U937 cells cultured without LPS (Figure 4C).

### TNF-α and IL-6 up-regulate Tn expression in HGFs

**Figure 5:**
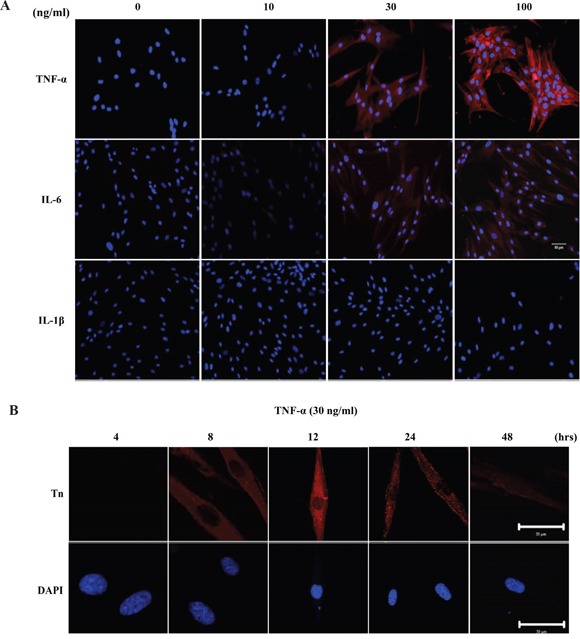
Pro-inflammatory cytokines, TNF-α and IL-6, up-regulates Tn levels in HGFs **A.** The effect of pro-inflammatory cytokines on Tn expression in HGFs. HGFs were treated with purified TNF-α, IL-6 and IL-1β at the concentration of 0, 10, 30, and 100 ng/ml for 24 hours and then stained with purified rabbit anti-Tn antibody (in red) and DAPI (in blue). Original magnifications (100x); scale bar, 50 μm **B.** Time course analysis of Tn expressions in HGFs after TNF-α treatment. HGFs were treated with purified TNF-α at the concentration of 30 ng/ml for 4, 8, 12, 24 and 48 hours and then stained with purified rabbit anti-Tn antibody (in red) and DAPI (in blue) (Magnifications 630x; scale bar, 50 μm). The experiments were repeated at least three times.

To determine whether cytokine(s) can elevate Tn levels, HGFs were treated with various amounts of purified cytokines. As shown in Figure 5A, Tn levels in HGFs were most responsive to TNF-α, moderately responsive to IL-6, and not responsive to IL-1β, even at a concentration of 100 ng/ml under the experimental conditions. Elevation of Tn by TNF-α (30 ng/ml) was shown to be time dependent. Tn levels in HGFs were essentially unchanged upon 4 hrs of TNF-α treatment. Gradual increase in Tn levels was observed between 8 to 12 hrs. The level of Tn gradually decreased after 24 hours of TNF-α treatment and markedly decreased after 48 hours of TNF-α treatment (Figure 5B).

### TNF-α up-regulates Tn expression through down-regulation of the *COSMC* gene

**Figure 6:**
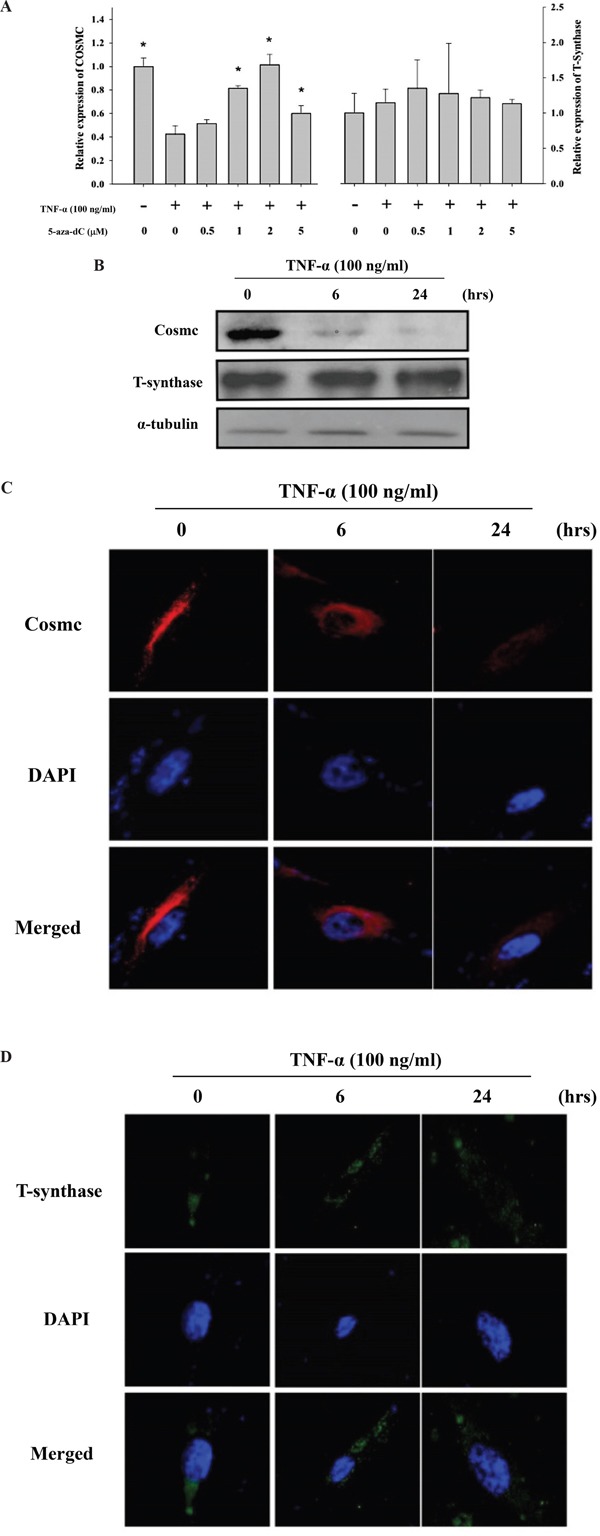
TNF-α up-regulated Tn levels is through down-regulation of the *COSMC* gene in HGFs **A.** Effect of TNF-α and demethylation agents (5-aza-dC) on mRNA expression of *COSMC* and *T-synthase* in HGFs. qPCR was used for analyze *COSMC* and *T-synthase* mRNA expression in HGFs upon TNF-α and 5-aza-dC treatment. The mRNA expression of *COSMC* and *T-synthase* was normalized to *GAPDH* and statistical analyzed. (*: p < 0.05 compared with TNF-α only). Calculations of relative gene expression (normalized to *GAPDH* reference gene) were performed according to the ΔΔCT method. Fidelity of the PCR reaction was determined by melting temperature analysis. **B.** Western blotting was used to analyze the protein levels of Cosmc and T-synthase upon 6 and 24 hours of TNF-α treatment. **C.** and **D.** HGFs were treated with purified TNF-α for 6 or 24 hours and then immunofluorescent stained with anti-Cosmc (in red) and anti-T-synthase antibodies (in green) and DAPI (in blue). Original magnifications (100x); scale bar, 50 μm.

**Figure 7:**
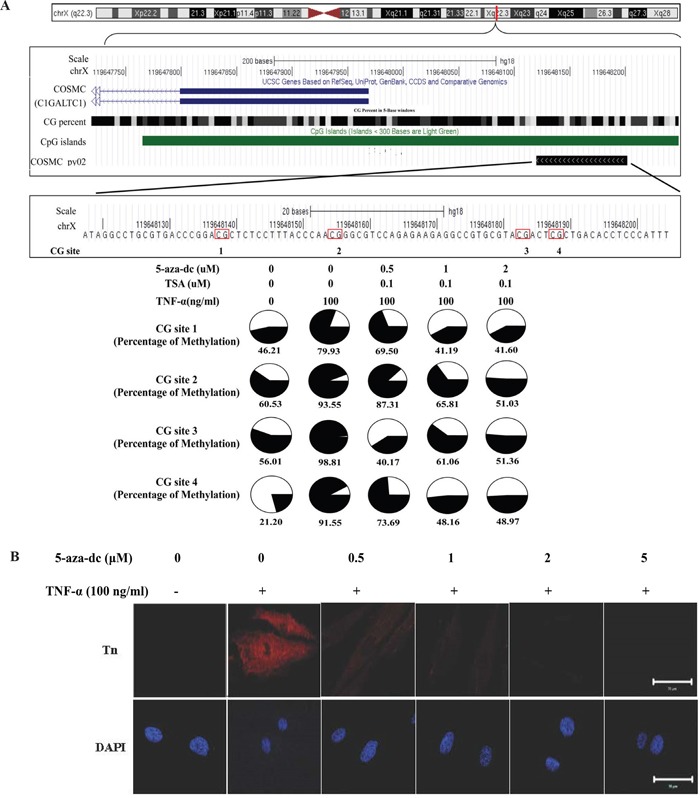
TNF-α-induced *COSMC* gene hypermethylation and Tn expression can be suppressed by demethylation agents (5-aza-dC) **A.** Effect of TNF-α and demethylation agents on the methylation level of *COSMC* gene in HGFs. The comparison of methylation changes is in the HGFs which were treated with or without TNF-α and demethylation agents. The UCSC genome browser illustrated the orientation and the first exon of *COSMC* (blue bar), GC percent (black scale bar), CpG islands (green bar) and the sequencing region of bisulfite pyrosequencing (COSMC_py02, black bar). The red circle shows CG islands, and the black color displays the level of methylation. **B.** HGFs were co-treated with purified TNF-α and different concentration of 5-aza-dC (0, 1, 2 and 5 μM) for 24 hours and then immunofluorescent stained with rabbit anti-Tn antibody (in red) and DAPI (in blue). Magnifications 630x; Scale bar, 50 μm.

To explore the possible molecular mechanism underlying cytokine-mediated up-regulation of Tn levels, the effect of TNF-α on the mRNA level of the *COSMC* gene was investigated. As shown in Figure 6A, TNF-α (100 ng/ml, treatment for 24 hrs) significantly down-regulated the *COSMC* mRNA in HGFs. By contrast, TNF-α did not significantly alter the T-synthase mRNA level. Similar results were observed for the protein levels of Cosmc and T-synthase in HGFs upon TNF-α treatment (Figure 6B, 6C and 6D). The effect of TNF-α on the down-regulation of the *COSMC* gene could possibly involve hypermethylation of the CpG islands in its promoter. Using bisulfite pyrosequencing to quantify the methylation change in the promoter of the *COSMC* gene, four *CpG* sites were significantly hypermethylated by TNF-α treatment (Figure 7A). Pretreatment of HGFs with demethylating agents decreased the methylation of the four *CpG* sites in the *COSMC* promoter in a dose-dependent manner (Figure 7A), and correspondingly, increased the expression of the *COSMC* mRNA and decreased the level of Tn (Figure 7B). In the aggregate, our results suggest that cytokine-mediated up-regulation of Tn levels is due to down-regulation of *COSMC*, which involves hypermethylation of the *COSMC* gene promoter.

## DISCUSSION

In the present studies, we have explored the molecular mechanism for elevated expression of the Tn antigen in cancer and inflammation. Our results show that oncogenic *Hras^G12V^*-transformed BEAS-2B cells express higher levels of Tn than untransformed BEAS-2B cells, suggesting a positive regulatory role of oncogenic Ras on Tn expression. Furthermore, conditioned media from oncogene-transformed and cancer cells were shown to stimulate Tn expression in freshly cultured recipient cells. Similarly, conditioned media from LPS-treated U937 cells were shown to elevate Tn expression in recipient cells, suggesting a potential role of cytokines or other soluble factors in regulating Tn expression. Indeed, proinflammatory cytokines TNF-α and IL-6 were shown to upregulate Tn expression in cultured gingival fibroblasts. Together, these results suggest that proinflammatory cytokines such as TNF-α and IL-6 could represent one regulatory pathway connecting oncogenic Ras to Tn expression.

Up-regulation of Tn expression by TNF-α and IL-6 could be the result of NF-κB (nuclear factor kappa-light-chain-enhancer of activated B cells) activation since NF-κB is known to increase the expression of a large number of cytokines including TNF-α and IL-6 [24, 25]. Our observation that oncogenic Ras up-regulates Tn expression could be explained by Ras-induced NF-κB activation which are known to regulate the expression of proinflammatory cytokines TNF-α and IL-6 [26, 27]. Indeed, previous studies have demonstrated that the expression of both Tn and NF-κB is correlated positively with staging, recurrence, distant metastasis, and invasive pattern grading score (IPGS) in oral squamous cell carcinoma (OSCC) [16]. Since constitutive activation of NF-κB is commonly observed in tumors [28–31], it seems possible that elevated Tn expression in tumors is at least in part due to NF-κB activation. It is also known that the interaction between stromal cells and tumor cells further enhances cytokine expression in the tumor microenvironment [32, 33]. Elevated Tn expression may thus be a potential marker for the tumor microenvironment. The strong correlation between the degree of tumor malignancy and Tn expression [34] could support such a view.

Our results also suggest a potential molecular mechanism for up-regulation of Tn by proinflammatory cytokines TNF-α and IL-6. Previous studies have demonstrated that Cosmc, a chaperon required for T-synthase folding and activity is often mutated in tumors [35]. The loss of function of Cosmc results in inactivation of T-synthase and hence elevated Tn expression. Consequently, the proinflammatory cytokines TNF-α and IL-6 could up-regulate Tn expression through the cytokine-mediated down-regulation of Cosmc.

Tn is known to be further glycosylated by several glycosyltransferases, T-synthase being only one of them [36–38]. Inactivation of T-synthase due to down-regulation of Cosmc may represent only one such mechanism leading to Tn up-regulation. For example, other glycosyltransferases, which transfer a monosaccharide such as sialic acid and N-acetyl-glucosamine (GlcNAc) to the Tn antigen may also be inactivated by proinflammatory cytokines through Cosmc-independent mechanisms. It remains to be determined whether these glycosyltransferases are also involved in cytokine-mediated Tn up-regulation.

The role of Cosmc in Tn up-regulation in tumors has been investigated [6, 39–42]. It has been shown that elevated Tn levels in tumors can result from loss-of-function mutations and loss of heterozygosity of the *COSMC* gene [43]. In addition, patients with Tn syndrome, an autoimmune disease, have acquired somatic mutations in the open reading frame of the *COSMC* gene in the hematopoietic stem cells [13, 44]. Collectively, mutations in the *COSMC* gene may be one of the mechanisms responsible for elevated Tn levels in tumors. Our current results could provide another mechanism for *COSMC* gene inactivation in tumors through tumor microenvironment-mediated epigenetic regulation of the *COSMC* gene.

Our current results showing epigenetic regulation of the *COSMC* gene (e.g. rescue by 5-aza-dC) are also consistent with previous reports that the T-synthase activity can be rescued after treatment of Tn-positive T cells from Tn syndrome patients with the DNA methylation inhibitor 5-azacytidine or the HDAC inhibitor sodium n-butyrate [45, 46]. In addition, the *COSMC* gene in Tn-positive B lymphocytes derived from the leukocytes of a male Tn patient was shown to epigenetically silenced through its promoter hyper-methylation. In this case, the *COSMC* gene transcription and T-synthase activity were also recovered by the 5-aza-dC treatment [47]. Together, these studies strongly suggest that, in addition to gene mutations, epigenetic regulation of the *COSMC* gene may represent an important mechanism for *COSMC* gene inactivation in tumors. In view of the increasing importance of Tn and related tumor-associated carbohydrate antigens in cancer immunotherapy, further studies of the molecular basis for tumor microenvironment-mediated *COSMC* gene inactivation seems warranted.

## MATERIALS AND METHODS

### Cell culture and treatment

BEAS-2B, an immotalized human bronchial epithelial cell line [48] and *Hras^G12V^*-transformed-BEAS-2B cells which stably expressing mutant *H-ras* (*Hras^G12V^*) were kindly provided by Dr. Ling-Huei Yih. Primary human gingival fibroblasts (HGFs) were cultured from clinically healthy male gingival tissue obtained from an individual undergoing crown-lengthening surgery. The protocol was approved by the Institutional Review Board of the Tri-Service General Hospital, National Defense Medical Center, Taipei, Taiwan (TSGHIRB No. 1-101-05-121), and informed patient consent was obtained. In all experiments, HGFs from the second passage were used. BEAS-2B, *Hras^G12V^*-transformed-BEAS-2B cells, HGFs and U937 cells were maintained in DMEM (Gibco) supplemented with 10% (v/v) fetal bovine serum (Gibco), glutamine 2 mmol/l (Gibco), penicillin (100 U/ml) (Gibco) and streptomycin (100 U/ml) (Gibco). For conditioned medium treatment, HGFs were prepared on chamber slides and then treated with conditioned medium obtained from U937 cells stimulated with various concentrations of *Escherichia coli* LPS (0, 10, 30, or 100 ng/ml; InvivoGen, San Diego, CA, USA) for 24 hours. For TNF-α, 5-Aza-2′-deoxycytidine (5-aza-dC) and trichostatin A (TSA) treatment, HGFs seeded in chamber slides or cell culture dishes were pretreated with various concentrations of 5-aza-dC (0, 0.5, 1, 2, and 5 μM; Sigma-Aldrich.) and/or trichostatin A (TSA, 0.1 μM; Sigma-Aldrich.) and then washed with PBS for three times and treated with TNF-α (100 ng/ml; Sigma-Aldrich.) for 24 hours. Trichostatin A, a histone deacetylases (HDAC) inhibitor, can relax chromatin and enhance the effect of demethylation by 5-aza-dC by repressing HDAC activity [49, 50].

### Specimens

Tissue sections of human breast (from 26 patients), prostate (from 30 patients) and cervical cancers (from 3 patients) were kindly provided by Dr. Nieh and the protocol was approved by the Institutional Review Board of the Tri-Service General Hospital, National Defense Medical Center (TSGHIRB No. 100-05-032). The methods of inducing atherosclerosis [51], bronchitis [52] and periodontotitis [53] are described previously.

### Immunohistochemistry

Tissue sections were dewaxed in xylene (Sigma-Aldrich.) and rehydrated in alcohol (Sigma-Aldrich.). Antigen retrieval was carried out by incubating tissue sections in citrate antigen retrieval buffer (10 mM citric acid, 0.05% Tween 20, pH 6.0) at 95°C for 40 minutes in a water bath. Endogenous peroxidase was blocked with 0.3% hydrogen peroxide (Sigma-Aldrich.) for 30 minutes. Tissue sections were then incubated with 5% normal horse serum (Gibco) in PBS for 30 minutes at room temperature in order to block nonspecific antibody reaction. After washing with TBS (0.05 M Tris-HCl, 0.9% NaCl, pH 8.4) plus 0.1 % Tween 20, slides were incubated for 30 mins at room temperature with mouse monoclonal anti-Tn antibody obtained from ascites (homemade; [16, 54]). Tissue sections were then rinsed in TBS plus 0.1% Tween 20 and incubated for 20 min at room temperature with peroxidase labelled polymer conjugated anti-mouse IgG (H+L) (DAKO, Denmark). Subsequently, tissue sections were stained with liquid DAB+ substrate-chromogen solution (DAKO, Denmark), counterstained with Mayer's hematoxylin, dehydrated, and mounted.

### Immunofluorescent staining and confocal laser scanning microscopy

Cells were fixed with 4% paraformaldehyde (Sigma-Aldrich.), blocked with 1% bovine serum albumin (Sigma-Aldrich.) in PBS for 1 hour, and then incubated with the purified rabbit anti-Tn antibody (homemade; purified from rabbit sera [16, 54]), mouse monoclonal anti-Tn antibody from ascites (homemade; [16, 54]), rabbit polyclonal anti-Cosmc (Genetex, N3C3) or mouse monoclonal anti-T-synthase (Abcam, ab57492) antibodies overnight at 4°C. The cells were washed six times in PBS and incubated with the secondary antibody [Texas Red-conjugated goat anti-rabbit IgG or 1:500; FITC-conjugated goat anti-mouse IgG, 1:500 (Sigma-Aldrich.)] for 1 hour and then counterstained with DAPI. The levels of Tn in HGFs was observed by confocal laser scanning microscopy (LSM780, Carl Zeiss MicroImaging, Inc., Thornwood, NY, USA).

### Enzyme-linked immunosorbent assay (ELISA)

The protein concentrations of TNF-α, IL-6 and IL-1β secreted into the media obtained from U937 cells treated with LPS (0, 10, 30 and 100 ng/ml) for 24 hours were measured. All media were collected, and the concentrations of cytokines were measured by commercial ELISA kits (R&D Systems, Minneapolis, MN) according to the manufacturer's protocols. A multiplate reader (Thermo Fisher Scientific) was set to measure the absorbance at wavelengths of 450 and 540 nm.

### Quantitative real-time PCR

To examine mRNA expression, we treated HGFs with or without TNF-α (100 ng/ml) and 5-aza-dC (0.5, 1, 2, 5 μM). Total RNA was extracted with TRIsure reagent (Bioline Ltd., London, United Kingdom). To eliminate the contamination of genomic DNA, total RNA was treated with DNase I (Life Technologies Corporation, Carlsbad, California, USA) before first strand cDNA synthesis, according to the manufacturer's instructions. One μg of DNase I-treated total RNA was reverse-transcribed with Tetro RT enzyme (Bioline Ltd.) into cDNA, and used as the template for real-time PCR reactions and analysis. The real time PCR reactions were performed using Rotor-GeneTM SYBR^®^ Green PCR Kit on Rotor-Gene Q (QIAGEN, Hilden, Germany). Transcribed cDNA was amplified using QuantiTect Primer Assay gene expression assay, including QT00494410 for human core 1 β3-Gal-T-specific molecular chaperone (*COSMC*), QT00045976 for human core 1 synthase, core 1 β1-3-galactosyltransferase (*C1GALT-1*, *T-synthase*), and QT01192646 for the endogenous control glyceraldehyde-3-phosphate dehydrogenase (*GAPDH*). This involved an initial denaturation at 95°C for 5 min, followed by 40 cycles of denaturing at 95°C for 5 sec and combined annealing/extension at 60C for 10 sec, as described in the manufacturer's instructions. Calculations of relative gene expression (normalized to *GAPDH* reference gene) were performed according to the ΔΔCT method [55]. Fidelity of the PCR reaction was determined by melting temperature analysis. All of our PCR experiments were done in triplicates and some of the experiments were repeated several times.

### Western blotting

To determine the protein expression of Cosmc and T-synthase, HGFs were treated with or without TNF-α (100 ng/ml). Whole cell lysates (30 μg) after denaturing under reducing conditions were separated on a 12.5 % SDS-PAGE, and transferred to PVDF membrane in transfer buffer (500 mM glycine, 50 mM Tris-HCl, 0.01% SDS, 20% methanol) at 350 mA for 1.5 h. PVDF membrane was washed in TBS-T (10 mM Tris-HCl, 100 mM NaCl, 0.1% Tween at pH 7.4) and then blocked with 5% non-fat milk extract in TBS-T for 30 min. The membrane was first blotted with rabbit polyclonal anti-Cosmc (Genetex, N3C3), mouse monoclonal anti-T-synthase (Abcam, ab57492) and rabbit polyclonal anti-α tubulin antibodies (Abcam, San Diego, CA), respectively and then blotted with HRP-conjugated anti-mouse or anti-rabbit IgG antibodies (Santa Cruz). After washing the membrane three times for ten minutes, signals were visualized using ECL substrate kit (Thermo science).

### Statistical analysis

Independent *t* tests were used to determine the effects of pretreatment with 5-aza-dC on TNF-α-induced *COSMC* and *T-synthase* mRNA expression.

### Bisulfite conversion

One microgram of genomic DNA was bisulfite converted using the CpGenome Fast DNA Modification Kit (Chemicon-Millipore, Billerica, MA, USA) according to the manufacturer's recommendations and was eluted in 70 μl nuclease-free water.

### Bisulfite pyrosequencing

We used bisulfite pyrosequencing to investigate the DNA methylation levels. Bisulfite pyrosequencing was performed following the manufacturer's recommendation for the PyroMark Q24 instrument (Qiagen, GmbH, Hilden, Germany). The results are expressed as percentages. The bisulfite pyrosequencing primers were design by PyroMark Assay Design 2.0 software. The PCR products were amplified using a PyroMark PCR Kit (Qiagen) in a total volume of 20 μl. The PCR reaction contained 1.8 μl bisulfite-converted DNA, 500 nM of each primer, and 1 × PCR Master Mix under the following conditions: denaturation at 95°C for 15 minutes, 60 polymerization cycles (94°C for 45 seconds; 60°C for 30 seconds, and 72°C for 45 seconds), and final extension at 72°C for 10 minutes. The sequence of *COSMC* primers: forward primer, 5′-GGTTTAAGGAAGTAGGGGTATATTAGAGA; 5′end of biotinylated reward primer, 5′-CCCCCTACCCAATTCAAAAACTTAC and sequencing primer, 5′-ATGTTAGGAAGGTGAAA.
